# Malaria infection by sporozoite challenge induces high functional antibody titres against blood stage antigens after a DNA prime, poxvirus boost vaccination strategy in Rhesus macaques

**DOI:** 10.1186/1475-2875-10-29

**Published:** 2011-02-08

**Authors:** Muzamil Mahdi Abdel Hamid, Edmond J Remarque, Ibrahim M El Hassan, Ayman A Hussain, David L Narum, Alan W Thomas, Clemens HM Kocken, Walter R Weiss, Bart W Faber

**Affiliations:** 1 Department of Parasitology, Biomedical Primate Research Centre, Rijswijk, The Netherlands; 2 Institute of Endemic Diseases, University of Khartoum, Sudan; 3 Naval Medical Research Centre, Silver Spring, Maryland, USA; 4 Laboratory of Malaria Immunology and Vaccinology, National Institute of Allergy and Infectious Diseases, NIH, USA; 5 Faculty of Medicine, Jazan University, Kingdom of Saudi Arabia

## Abstract

**Background:**

A DNA prime, poxvirus (COPAK) boost vaccination regime with four antigens, i.e. a combination of two *Plasmodium knowlesi *sporozoite (*csp/ssp2*) and two blood stage (*ama1/msp1*_*42*_) genes, leads to self-limited parasitaemia in 60% of rhesus monkeys and survival from an otherwise lethal infection with *P. knowlesi*. In the present study, the role of the blood stage antigens in protection was studied in depth, focusing on antibody formation against the blood stage antigens and the functionality thereof.

**Methods:**

Rhesus macaques were immunized with the four-component vaccine and subsequently challenged i.v. with 100 *P. knowlesi *sporozoites. During immunization and challenge, antibody titres against the two blood stage antigens were determined, as well as the *in vitro *growth inhibition capacity of those antibodies. Antigen reversal experiments were performed to determine the relative contribution of antibodies against each of the two blood stage antigens to the inhibition.

**Results:**

After vaccination, PkAMA1 and PkMSP1_19 _antibody titres in vaccinated animals were low, which was reflected in low levels of inhibition by these antibodies as determined by *in vitro *inhibition assays. Interestingly, after sporozoite challenge antibody titres against blood stage antigens were boosted over 30-fold in both protected and not protected animals. The *in vitro *inhibition levels increased to high levels (median inhibitions of 59% and 56% at 6 mg/mL total IgG, respectively). As growth inhibition levels were not significantly different between protected and not protected animals, the ability to control infection appeared cannot be explained by GIA levels. Judged by *in vitro *antigen reversal growth inhibition assays, over 85% of the inhibitory activity of these antibodies was directed against PkAMA1.

**Conclusions:**

This is the first report that demonstrates that a DNA prime/poxvirus boost vaccination regimen induces low levels of malaria parasite growth inhibitory antibodies, which are boosted to high levels upon challenge. No association could, however, be established between the levels of inhibitory capacity *in vitro *and protection, either after vaccination or after challenge.

## Background

Malaria is a leading cause of morbidity and mortality affecting billions of people worldwide. It is estimated that malaria is responsible for the annual death of 800,000 people, mostly children under the age of five [1]. In the face of increasing resistance of *Plasmodium *parasites to anti-malarial (prophylactic) drugs, development of an effective malaria vaccine is generally considered a public health priority [2]. Feasibility of a successful malaria vaccine has been demonstrated by immunization with irradiated sporozoites and subsequent malaria infection in rodent, non-human primate and human models [3-5]. Furthermore, natural long-term exposure to the parasite is associated with an age-related decrease in the incidence, prevalence and density of infection [6].

The traditional approach for malaria vaccine development is based on recombinant proteins administered in combination with novel adjuvants, directed either to erythrocytic or pre-erythrocytic stages of the parasite. Early clinical trials conducted with the pre-erythrocytic particulate protein vaccine RTS,S showed moderate levels of efficacy [7]. Protein subunit vaccines do have a number of disadvantages. One is that they require the use of adjuvants that may induce to adverse effects and may be difficult to get access to, due to intellectual property rights. Moreover, antigen conformation and stability (with or without adjuvant) at ambient temperatures are also major issues that may complicate the use of subunit vaccines.

To circumvent these caveats, alternative vaccine delivery platforms have been developed. These include, among others, viral vector approaches, DNA vaccination and virosomal delivery systems, combinations of DNA and viral vector in prime-boost strategies, and protein/adjuvant booster strategies [8-13].

Previous studies with the malaria murine challenge model have shown that DNA vaccines encoding *Plasmodium *antigens are able to induce CD4+ and antibody responses, as well CD8+, CTL and IFNγ responses required to attack parasites as they develop inside hepatocytes [14-16]. Phase I/IIa clinical trials have established the safety, tolerability and immunogenicity of DNA vaccines encoding malaria parasite antigens in healthy individuals [2,17].

A DNA prime (3x), poxvirus (COPAK) boost (1x) vaccination regimen comprising two sporozoite (*csp/ssp2*) and two blood stage (*ama1/msp1*_*42*_) antigens (Pk4x3/COPAK) was developed at the Naval Medical Research Centre. This reproducibly yields high levels (>60%) of protection in the rhesus macaque/*Plasmodium knowlesi *sporozoite challenge model [12,18,19]. The immunological analysis of these studies [19] focused on the cellular immune response. The parameter measured (IFN-γ ELIspot) did not correlate with protection. It was noted that immunization with a similar vaccine, containing two sporozoite antigens (*csp/ssp2*), using the same immunization schedule, resulted in a one-day delay in the onset of parasitaemia, but not in protection. This delay was not accompanied by lower parasite growth rates in the blood stage, when compared to naive animals [19]. This suggested that protection is critically depended on the blood stage antigens included in the Pk4x3/COPAK vaccine. Therefore, in this study the titres and functionality of the antibodies from blood samples of the above studies (before and after challenge) were analysed using ELISA and *in vitro *growth inhibition assays. Subsequently, GIA inhibition levels (after vaccination and after challenge) were compared between protected and not protected animals, in order to establish potential correlates of protection.

## Methods

### Plasmid DNA vaccines and poxvirus

The DNA plasmid and COPAK poxvirus immunization vector (Virogenetics, Troy, N.Y) encoding two (*csp/ssp2*) or four *P. knowlesi *genes (*csp/ssp2/ama1/msp1*_*42*_) are previously described. COPAK is derived from the Copenhagen strain of vaccinia virus [12].

### Antigen preparation

PkMSP1_19 _was produced and purified as described previously [20]. PkAMA1 was expressed in *Pichia pastoris *and produced as described previously [21]. Briefly, a synthetic gene, comprising domain I-II-III of *P. knowlesi *H strain AMA1 (Accession code XM_002259303) and a hexa-histidine tag, codon optimized for expression in *Pichia pastoris *(DNA20, Menlo Park, CA), was cloned into the pPicZαA vector (Invitrogen, Leek, The Netherlands) and transformed into *P. pastoris *Km71H.

### Rhesus monkeys, immunization regimen and challenge

The immunization and challenge phase of these studies have been published, as well as the immunological analysis focusing on the sporozoite antigens [19]. Briefly, in these studies three vaccination groups were used: 1) a four antigen (Pk4) (*csp/ssp2/ama1/msp1*_*42*_) vaccine regimen, 2) a two antigen (*csp/ssp2*) vaccine regimen, and 3) a control vaccine (mock vaccine; an empty DNA plasmid and empty COPAK virus). The immunization regimen included a prime with three injections of DNA (dose of 0.5 mg of each plasmid in a volume of 1 mL) given at day 0, 28, 56. Four months later (day 168) the monkeys received a booster immunization with 2 ×10^8 ^pfu of COPAK virus, for each individual antigen [19].

Three weeks after the COPAK booster (day 189), pre-challenge blood samples were collected. One week later (day 196), animals were challenged by intravenous injection of 100 *P. knowlesi *(H strain) sporozoites [19]. Animals were termed 'protected' when able to control the parasitaemia below 1.5% and eventually to undetectable levels, after challenge. Animals unable to control the parasitaemia below 1.5% were treated with chloroquine [19] and were termed 'not protected'. A summary of the outcomes is presented in Table 1. Four weeks after challenge (day 224), a final blood sample was taken. At day 224, animals not yet treated were given chloroquine.

**Table 1:** Observed parasitaemia in Pk4 vaccinated, CSP/SSP2 vaccinated and control (mock vaccine) groups.

Experiment #	Vaccine group	Rhesus ID	**day1**^**st **^**parasitaemia**	**Mean 1**^**st **^**day parasitaemia**	day >1.5% parasitaemia	Mean day >1.5% parasitaemia
1	Mock*	20H	7	7.0	12	11.5
1	Mock	205	7		12	
1	Mock	AB07†	7		11	
1	Mock	AB58	7		12	
1	Mock	Q121	7		11	

2	csp/ssp2	IIG	9	8.2	11	11.2
2	csp/ssp2	AB67	8		11	
2	csp/ssp2	AC70	8		12	
2	csp/ssp2	Q134	8		10	
2	csp/ssp2	AK52	8		12	
2	csp/ssp2	IIG	9		11	

2	Pk4**	281	11	9.5	14	13
2	Pk4	284	9		13	
2	Pk4	3000	9		13	
2	Pk4	3129	9		13	
2	Pk4	19159	9		13	

2	Pk4	262	9	9.5	Never	Never
2	Pk4	299	10		Never	
2	Pk4	3086	11		Never	
2	Pk4	3098	9		Never	
2	Pk4	AB34	9		Never	
1	Pk4	Q120	9		Never	
1	Pk4	T152	10		Never	
3	Pk4	228	10		Never	

† Animal died before the end of the study. No post challenge sample was available.

* Mock, vaccine comprised of empty DNA vector and empty COPAK vaccinia virus

** Pk4, vaccine comprised of four *Plasmodium knowlesi *antigens, csp/ssp2/ama1/msp1_42_

Day to 1^st ^parasitaemia is the day of patency of parasites on thin smear. Day >1.5% parasitaemia is the day of drug treatment. All vaccinated animals were primed with 3 DNA vaccine injections and boosted with recombinant poxviruses encoding the Pk4 antigens. Control (mock) animals received mock DNA vaccine and empty poxvirus. Subjects were taken from three experiments (#1-3), under identical vaccination regimens.

### ELISA

ELISA's were performed in 96-well flat bottom micro titre plates (Greiner, Alphen a/d Rijn, The Netherlands), coated with either 0.5 μg mL^-1 ^of PkMSP1_19 _or PkAMA1 according to published methods [22]. Titres are expressed as arbitrary units (AU), where 1 AU yields an optical density of 1.0 over background. Thus, the AU-value of a sample is the reciprocal dilution at which the absorbance at 405 nm equals 1.0. All assays were performed in duplicate.

### IgG purification

Total IgG was isolated on protein A columns (Sigma, St. Louis, MO). Elution buffer was exchanged for RPMI 1640 by repeated concentration/dilution using Amicon Ultra-15 concentrators (30-kDa cutoff; Millipore BV, Amsterdam, The Netherlands). IgG fractions were filter sterilized and stored at -20°C until use. IgG concentrations were determined using a Nanodrop ND-1000 spectrophotometer (Nanodrop Technologies, Wilmington, DE).

### Parasite growth inhibition assay

The ability of protein A purified rhesus IgG to inhibit *in vitro *parasite growth was assessed in triplicate using 96-well flat-bottomed tissue culture plates (Greiner, Alphen a/d Rijn, The Netherlands) with *in vitro *matured and synchronized *P. knowlesi *(H strain) schizonts at a starting parasitaemia of 0.8-1.0%, a haematocrit of 2.0%, in RPMI 1640 fortified with 10% normal human serum and 20 μg mL^-1 ^gentamicin, in a final volume of 100 μL.

For antigen reversal GIA experiments, PkAMA1 and PkMSP1_19 _antigens were dialyzed against RPMI 1640, concentrated and filter-sterilized. Then they were serially diluted and incubated with isolated total rhesus IgG, at a concentration that was determined to result in an inhibition of 80% (in the absence of added antigens). Incubation was in incomplete culture medium (total volume 50 μL per well) for 45 min at room temperature, followed by 15 min of incubation at 37°C in a 96-well tissue culture plate. A parasite suspension containing schizonts in culture medium with 40% normal human serum was prepared and added to the plate to adjust the cultures to the same parasitaemia and haematocrit levels as used in the standard GIA described above.

After incubation of 24 to 26 hours, 30 μL of the resuspended culture was added to 200 μL ice-cold PBS, pH 7.4. After brief centrifugation, the supernatant was removed and pellets were frozen. Parasite lactate dehydrogenase levels were determined in the thawed pellets, as previously described [23]. From the pLDH levels, parasite growth inhibition reported as percentage was calculated as follows: 100-[(A_650 _of infected RBCs with test IgG - A_650 _of uninfected RBCs)/(A_650 _of infected RBCs with test IgG, at T = 0 - A_650 _uninfected RBCs, at T = 0) × 100]. All GIA and reversal GIA results reported are the averages of two independent GIA assays.

### Statistics

All statistical analyses were performed using R software version 2.8.1 (R foundation for Statistical Computing, Vienna, Austria). IgG titres were log-transformed to obtain normality and significance was assessed by t-tests; a correction for unequal variances was applied where necessary. IgG antibody levels are presented as geometric means with 95% confidence intervals. The statistical significance of changes in IgG titres between time points were assessed using a paired t-test and presented as a ratio with 95% confidence intervals. Between group comparisons of GIA titres were performed using Mann-Whitney U test and titres are presented as medians with quartile ranges. Changes in GIA titres between time points were assessed using a paired t-test and data are presented as a difference (in percent points) with the corresponding 95% confidence intervals. The relation between GIA titre and PkAMA1 or PkMSP1_19 _antibody levels was assessed by Spearman's rank correlation, the correlation is presented as Spearman's Rho. Two-sided P values less than 0.05 were considered significant.

## Results

This study is a further analysis of samples obtained during a vaccination and challenge study, published by Weiss and co-workers [19]. In that study, approximately 60% (8 out of 13) of monkeys were protected from challenge with *P. knowlesi*, after receiving three injections with DNA encoding 4 *Plasmodium knowlesi *antigens (*csp/ssp2/ama1/msp1*_*42*_) and a booster with a mixture of 4 COPAK viruses, encoding the same antigens. Monkeys receiving a similar vaccination regimen, but with DNA and poxvirus comprising sporozoite antigens (*csp/ssp2*) only, or mock vaccine, were not able to control the parasitaemia (not protected). A summary of the outcomes of this study is presented in Table 1.

### Antibody responses induced by DNA prime/viral boost vaccination

**Figure 1:**
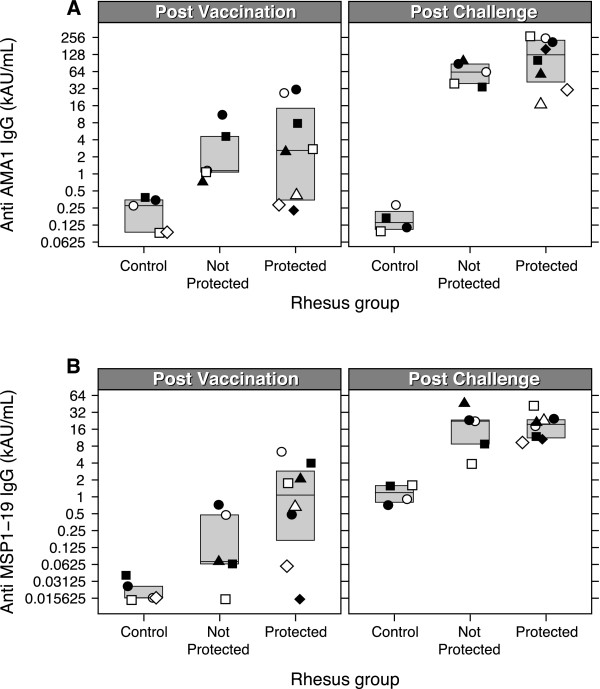
**ELISA titters against PkMSP1_19 _and PkAMA1 before and after challenge**. IgG antibody titres were measured by ELISA. The superimposed box around the data points indicate the upper and lower quartiles, the line in the middle indicates the median value. A) Antibody titres to PkMSP1_19 _in the control (mock vaccine receiving) animals and Pk4-vaccinated animals (protected or not protected) before challenge (left panel) and after challenge (right panel). B) Antibody titres to PkAMA1 in the control (mock vaccine receiving) animals and vaccinated animals (protected or not protected) before challenge (left panel) and after challenge (right panel). Geometric shapes represent individual animals in each group, throughout all figures. For one animal in the control group no post challenge data are available, as it died for study-unrelated reasons.

ELISA titres for the two blood stage antigens (PkMSP1_19_/PkAMA1) were determined in serum samples obtained from *csp/ssp2 *and *csp/ssp2/ama1/msp1*_*42 *_animals, both before and after challenge, and run in a single experiment (Figure 1, Table 2). Pre-immune sera were all negative. Following Pk4x3 prime and poxvirus (COPAK) booster vaccination, all monkeys seroconverted for both PkMSP1_19 _and PkAMA1 antigens. Geometric mean titre for PkMSP1_19 _was 332 AU/mL (95% CI: 97-1,136) while the titre for PkAMA1 appeared higher at 2,305 AU/mL (95% CI: 855-6,214).

**Table 2:** Geometric means of antibody titres as determined by ELISA.

	All PkAMA1	Protected PkAMA1	Not protected PkAMA1	**All PkMSP1**_**19**_	**Protected PkMSP1**_**19**_	**Not protected PkMSP1**_**19**_
Post vaccination	2,305 [855-6,214]	2,417 [469-12,451]	2,137 [508-8,990]	332 [97-1,136]	633 [109-3,671]	118 [16-853]

Post challenge	79,030 [46,803-133,444]	94,870 [40,055-224,699]	59,000 [32,980-105,548]	16,784 [11,104-25,369]	17,875 [11,730-27,240]	15,175 [4,562-50,477]

In brackets the 95% confidence intervals. No significant differences were observed between protected or not-protected groups of animals. For both antigens and all groups, antibody levels are significantly higher after challenge.

After vaccination, there were no significant differences between the PkMSP1_19 _and PkAMA1 antibody titres from protected and non-protected monkeys, respectively (T-test, P = 0.133 and P = 0.889, for PkMSP1_19 _and PkAMA1, respectively) (Figure 1, Table 2).

Four weeks after challenge, antibody levels to PkAMA1 and PkMSP1_19 _were boosted significantly. For PkAMA1 the antibody level was 34 fold [95% CI: 15-80, P = 9.9 e^-7^] higher post-challenge compared to pre-challenge. For PkMSP1_19 _the increase in the antibody titre after challenge was 50 fold [95% CI: 17-149, P = 4.2 e^-6^]. Again no significant differences were observed between protected and not protected animals (PkMSP1_19_; P = 0.94/PkAMA1; P = 0.35, t-test) (Figure 1, Table 2).

After challenge, control animals (mock-vaccinated) showed elevated antibody levels against PkMSP1_19_, while no increase in anti-PkAMA1 levels was observed. Before challenge, antibodies against PkAMA1 and PkMSP1_19 _were not detected in these animals (Figure 1).

### Functionality of the antibodies

Growth inhibition assays were performed to assess the functionality of the anti-blood stage antigen antibodies. Plasma samples from 13 animals were selected from previous Pk4 vaccination/challenge experiments with identical vaccination regimens [19]. These were divided into two groups, comprised of protected (N = 8) or not protected animals (N = 5). Samples from animals that received a mock vaccine (N = 5) and from animals that received *csp/ssp2 *vaccine (N = 5) were also included in the analysis (Table 1).

**Figure 2:**
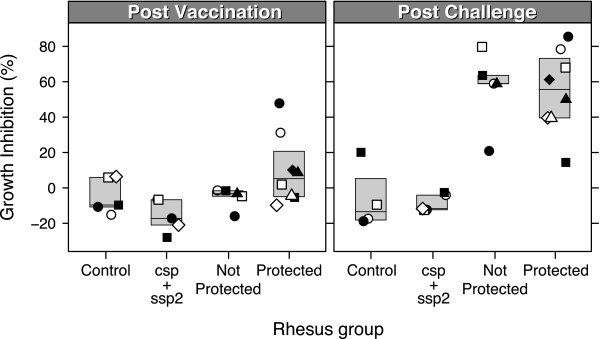
**Parasite growth inhibition activity of protected and non-protected monkeys**. A) Post vaccination B) post challenge. Mean GIA inhibition levels of total IgG isolated from monkey serum from animals in control (mock) group, CSP/SSP2 group, and Pk4x3/COPAK vaccinated animals (protected or not protected animals are shown). Final IgG concentration added to *P. knowlesi *parasite culture was 6 mg/mL. Geometric shapes represent individual animals in each group, throughout all figures. For one animal in the control group no post challenge data are available, as it died for study-unrelated reasons.

Figure 2 shows the results of parasite growth inhibition assays after vaccination (Panel A) and after challenge (Panel B) of Pk4-vaccinated animals, csp/ssp2-vaccinated animals and controls.

After vaccination, total IgG isolated from serum of protected animals inhibited growth of *P. knowlesi *between -10 and 48% (median inhibition 5.3 at 6 mg/mL IgG concentration). The animals with the highest growth inhibition levels (31% and 48% at 6 mg/mL, circles in Figure 2) were able to control the infection. Virtually no growth inhibitory antibodies were present in total IgG fractions isolated from plasma samples from animals that were not protected (Figure 2). No inhibition was observed using purified total IgG from plasma samples from animals receiving the sporozoite antigen vaccine or mock vaccine.

Four weeks after sporozoite challenge, a significant increase of 51% ([95% CI: 41-61%], P = 1.1 e^-7^; paired t-test) in the level of inhibition was observed in all Pk4 vaccinated animals (Figure 2). Purified total IgG isolated from plasma samples from protected animals inhibited parasite growth ranging from 14% to 80% (median inhibition of 56% at 6 mg/mL IgG concentration), while not protected animals had inhibition levels ranging from 21 to 80% (median inhibition of 59%).

GIA levels between protected and not protected animals were not significantly different (t-test), either before (P = 0.07234) or after challenge (*P *= 0.8884). This suggests that there is also no association between GIA levels and protection.

Other studies have shown a positive correlation between antibody levels and GIA inhibition (f.e. [24]). In this study this correlation was confirmed, by Spearman's rank correlation test (for PkMSP1_19_: rho = 0.683, P = 3.5 e^-6^; for PkAMA1: rho = 0.754, P = 8.7 e^-7^).

Purified total IgG from CSP/SSP2 vaccinated monkeys and mock-vaccinated monkeys did not show any inhibition to *P. knowlesi *parasites *in vitro*, either before or after challenge (Figure 2).

### Specificity of the parasite growth inhibitory antibodies

**Figure 3:**
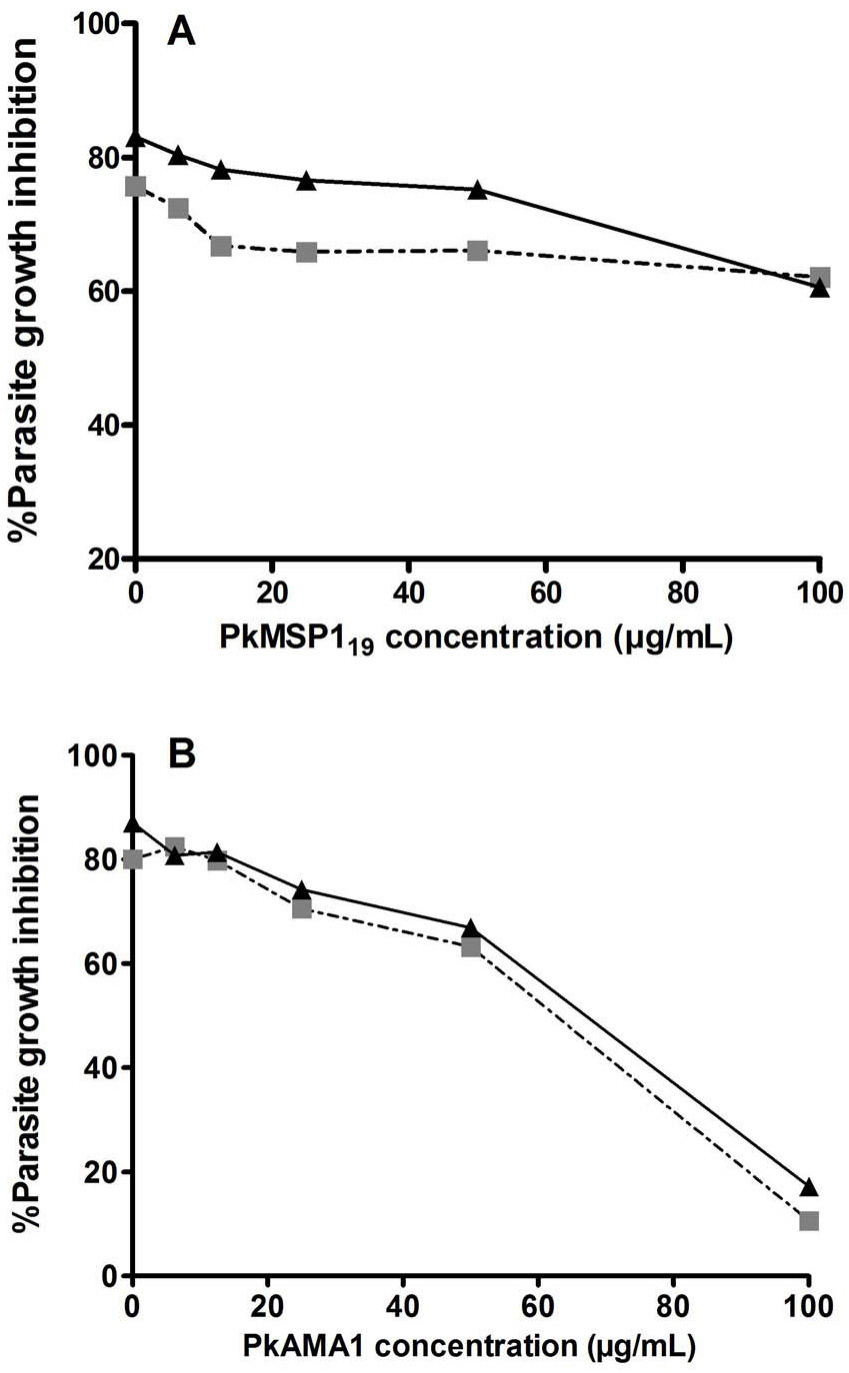
**Reversal of growth inhibitory activity by PkAMA1 and PkMSP1_19_**. Purified IgG from monkey 3086 (protected), post-challenge (black triangle). Mixture of pool purified IgG taken from Pk4-vaccinated monkeys (262, 299, 3086, 3098, AB34, Q120, T152, 228), post-challenge (black square). IgG was pre-incubated with either PkMSP1_19 _(Panel A) or PkAMA1 (Panel B) in a five-fold serial dilution, prior to mixing with *P. knowlesi *parasites. Results shown are the mean of two independent assays.

The ability of PkAMA1 and PkMSP1_19 _proteins to reverse growth inhibition was evaluated in the *in vitro *assay, in order to analyse the specificity of antibodies induced by DNA prime, poxvirus boost vaccination and *P. knowlesi *challenge. This was done using a pool of total IgG isolated from plasma of protected animals obtained after challenge and total IgG of a single protected animal (3086), also only after challenge. Antibodies were pre-incubated with increasing concentrations of PkAMA1 or PkMSP1_19_, prior to addition to a growth inhibition assay. Growth inhibition could be reversed to over 85% by addition of the PkAMA1 antigen at 100 μg/mL (Figure 3A). PkMSP1_19 _protein, at the same concentration, could only reverse inhibition by 10% (Figure 3B). The same degree of inhibition was obtained with post-challenge IgG isolated from a plasma sample of a single protected monkey (Figure 3). These results show that in this assay the larger part of the inhibitory activity is mediated by anti-PkAMA1 antibodies.

## Discussion and conclusions

A DNA prime, poxvirus boost vaccination regimen that has been shown to protect a high percentage of macaques from potentially lethal parasitaemia after *P. knowlesi *challenge [12,18,19] was further investigated. An important question left open from these studies was the nature of the immune response responsible for protection. Of the four malaria antigens included in the Pk4 vaccine, CSP and SSP2 are expressed on sporozoites and may be present in the early stage infected liver cells [25,26]. Although AMA1 and MSP1 are generally considered to be blood stage antigens [27,28], expression for both antigens in late liver schizonts has been demonstrated [29,30], while AMA1 is also expressed on the sporozoite surface membrane [29]. Thus, both sets of antigens can be considered to have added value as multi-stage vaccine candidates.

Rhesus monkeys receiving *csp *and *ssp2 *only demonstrated a delay in appearance of parasites in the blood (>1 day), but were not able to control the infection below 1.5% parasitaemia [19]. By contrast, monkeys that received the Pk4x3/COPAK vaccine including *ama1 *and *msp1*_*42*_, showed a significant delay in the appearance of parasites in the blood (>2 days) and 60% of these monkeys could control the infection below 1.5% parasitaemia. This is a strong indication that the immune response to the blood stage antigens was necessary for controlling parasite growth.

The cellular immune responses measured in the original study [19] did not correlate with protection. As antibodies are generally believed to be the key mediators for protection against blood stage malaria, the antibody responses to PkAMA1 and PkMSP1_19 _after vaccination were determined and found to be present at low levels, with corresponding low growth inhibition activity (median inhibition 5.3% in protected animals versus -3.3% in not protected animals). Challenge of the animals initially resulted in high parasite growth rates, for all animals. Obviously, *in vivo *parasite growth was not or only marginally inhibited. This is supported by the observation that functional antibody levels after vaccination, as determined in GIA, are low.

Although antibody levels against PkMSP1_19 _and PkAMA1 appear to be higher in protected animals compared to those in not protected animals (Figure 1), the differences are not statistically significant, likely to be the result of the low values and corresponding high variance of the data. Similarly, after vaccination there was no correlation between the (low) GIA levels and protection. Low GIA values (<15% inhibition) are difficult to interpret. For low inhibitions the two terms in the upper part of the equation used to calculate the inhibition (See methods), are relatively large and nearly equal to each other. A small deviation in either the control or the sample value will have a strong impact on the magnitude of the inhibition. The variance in the outcomes of the growth experiments is also one of the reasons why negative values are frequently observed in the GIA.

Sporozoite challenge boosted the levels of antibodies against PkMSP1_19 _and PkAMA1 in animals receiving the Pk4 vaccines over 30 fold, but not in monkeys receiving mock vaccines (Figure 1), supportive for the hypothesis that DNA prime followed by a poxvirus boost induces T-cell responses to the vaccine antigens. The induced CD4 T-cells provide help to B-cells upon (re-)exposure to the vaccine antigens, which may explain the increase in PkAMA1 and PkMSP1_19 _specific antibodies during challenge.

Although antibody levels were significantly elevated post challenge, no significant difference was observed between the antibody levels of protected and not protected animals, either for PkAMA1 or for PkMSP1_19_. Similarly, GIA inhibitions were not significantly different between protected and not protected animals after challenge (Figure 2).

The measured antibody titres and inhibition levels (GIA), four weeks after challenge are lower than the levels reported for PkAMA1/adjuvant immunization studies [21]. In these studies, even at high inhibition levels (~70% inhibition at 6 mg/mL total IgG) some animals were not able to control the infection. This is an indication that immune responses other than antibodies are likely to be involved in protection.

Interestingly, PkMSP1_19 _antibody levels were detected after challenge of naïve monkeys, while PkAMA1 antibodies were not (Figure 1). As in naturally exposed humans in endemic areas the anti-PfAMA1 antibodies titres are normally higher than those against PfMSP1_19_, [31,32], this may be explained by assuming that this is the result of a single exposure to the parasite, reflecting the difference in abundance of MSP1 and AMA1 on the parasite's surface, MSP1 being the most abundant protein on the merozoite surface, while AMA1 is poorly abundant.

In Figure 2, the values for the growth inhibition are negative for most groups, indicative for a small stimulation of growth in the presence of the antibodies that are added. This observation is not uncommon, and, as explained above, is related to the calculation of growth inhibition.

Almost complete reversal of the growth inhibition can be achieved by addition of 100 μg/mL PkAMA1 protein in the GIA assay. Titration of PkMSP1_19 _at the same concentration of protein leads to approximately 10% reversal of inhibition (Figure 3), implying that the larger proportion of the (GIA) inhibitory antibodies in the sera are directed against PkAMA1 rather than PkMSP1_19_. This observation does not necessarily imply that anti-PkMSP1_19 _antibodies do not contribute to the ability to control the infection. It is known that several mechanisms of antibody-mediated inhibition exist, some with the aid of immune cells (such as antibody dependent cellular inhibition (ADCI) [33]) that will not give a response in GIA. For anti-PfMSP1_19 _antibodies, it has been shown that Fc-tail mediated antibody responses may be important for protection in a humanized mouse model [34]. Moreover, part of the anti-MSP1_19 _antibodies may be growth-inhibitory rather than invasion inhibitory [35]. These mechanisms may result in an underestimating of the inhibitory capacity of the anti PkMSP1_19 _antibodies.

It has to be noted that all analyses of the response against the PkMSP1_42 _part of the vaccine was done using PkMSP1_19 _protein. It cannot be excluded that this may have lead to an underestimation of the antibody titres and of the ability to reverse the inhibition in the GIA reversal experiments. However, a protein/adjuvant immunization study with PfMSP1_42 _in humans has shown that the antibody response against PfMSP1_42 _is strongly directed to the PfMSP1_19 _part [36], warranting the conclusion that the main part of the GIA activity is directed against PkAMA1.

The present study could not establish a correlation between the levels of inhibitory antibodies, either before or after challenge, with the ability to control the infection. Previous analysis of these experiments has shown that also no correlation could be established between T-cell mediated immunity (ELIspot antigen-induced IFN-γ production), and protection [19].

The boost in functional antibody levels observed after sporozoite challenge is very interesting, especially in relation with the observed course of parasitaemia in the Pk4x3/COPAK vaccinated animals [19]. Boosting of the antibody levels will, per definition, take place after the parasites emerge from the liver and enter the circulation, as only then the antigens will be "visible" for the immune system. As the immune response against the blood stage antigens is the determining factor for protection in this model, it can be imagined that the growth rate of the parasites versus the increase over time in inhibiting capacity of the immune system, irrespective of the exact nature of this inhibiting capacity, determines whether an animal will be protected or not. Obviously, in protected animals the immune response is increasing in magnitude over time, while the parasitaemia increases to levels very near to 1.0%. The day the animals become patent with parasites (day 11/12) parasites are multiplying with a multiplication factor higher than one, resulting in higher parasitaemia the next day. Obviously the inhibitory capacity of the immune system is not high enough to arrest parasite multiplication. After two to three days, in the protected animals parasite levels become more or less constant, at 1% parasitaemia, and it can be argued that the animals' immune system has been built up to an extent that the inhibition equals the multiplication rate. In most protected animals this situation is maintained for a number of days, after which the parasites are cleared from the circulation. In non-protected animals the immune system is obviously not able to catch up with the growth of parasites. As *P. knowlesi *has a multiplication rate of, on average, 10 per 24 hours (one cycle) [37] (i.e. each ruptured schizont gives rise to 10 freshly infected RBCs), a value confirmed by the parasitaemia profile of the controls (Figure 1A, in [19]), the inhibition level at this point has to be close to 90%. For some animals, protected or not, the levels of functional antibodies, determined four weeks after challenge, are not too far off of this value. This shows that antibody levels may be of key importance for protection for some animals, but given the low inhibition values of other protected monkeys, other immunological responses may be of key importance for protection in these animals. A final note, something that has not been appreciated so far, is that the kinetics of the immune response may be of great importance for the outcome of an infection. The time it takes to reach the required inhibition levels, in relation to the course of the infection, may be as important as the final magnitude. Frequent, daily sampling (starting at the day of challenge) may reveal whether (functional) antibody production rates versus the course of the parasitaemia is correlated with protection. Another interesting possibility would be a second challenge, four weeks after the first, to investigate whether the high levels of antibodies that are present at that time may lead to protection of animals with high functional antibody titres.

Importantly, this study shows that in a vaccination regimen that is not focused on the production of antibodies, these are produced and their levels are significantly boosted after sporozoite challenge. These antibodies may play a direct role in protection alongside the cellular and antibody-mediated cellular immunity induced after vaccination and challenge.

The above studies show that the *P. knowlesi*-rhesus macaque challenge model could be instrumental for the eventual elucidation of factors that contribute to protection upon challenge with *P. knowlesi*.

## Competing interests

The authors declare that they have no competing interests.

## Authors' contributions

MMaH, BF, ER and WW made substantial contributions to the conception and the design of the study. MMaH did the work in lab, analysis and interpretation of data was done by MMaH, IH, AH, WW, ER and BF. MMaH drafted the manuscript, DN, AT, CK, ER, WW and BF revising it critically. MMaH, ER and BF gave final approval of the version to be published; all authors read and approved the final manuscript.
